# An updating-based working memory load alters the dynamics of eye movements but not their spatial extent during free viewing of natural scenes

**DOI:** 10.3758/s13414-023-02741-1

**Published:** 2023-07-19

**Authors:** Nicholas J. Wyche, Mark Edwards, Stephanie C. Goodhew

**Affiliations:** Research School of Psychology (Building 39), The Australian National University, Canberra, ACT 2601 Australia

**Keywords:** Visual attention, Working memory, Attentional breadth, Eye movements, Updating, Scene-viewing

## Abstract

The relationship between spatial deployments of attention and working memory load is an important topic of study, with clear implications for real-world tasks such as driving. Previous research has generally shown that attentional breadth broadens under higher load, while exploratory eye-movement behaviour also appears to change with increasing load. However, relatively little research has compared the effects of working memory load on different kinds of spatial deployment, especially in conditions that require updating of the contents of working memory rather than simple retrieval. The present study undertook such a comparison by measuring participants’ attentional breadth (via an undirected Navon task) and their exploratory eye-movement behaviour (a free-viewing recall task) under low and high updating working memory loads. While spatial aspects of task performance (attentional breadth, and peripheral extent of image exploration in the free-viewing task) were unaffected by the load manipulation, the exploratory dynamics of the free-viewing task (including fixation durations and scan-path lengths) changed under increasing load. These findings suggest that temporal dynamics, rather than the spatial extent of exploration, are the primary mechanism affected by working memory load during the spatial deployment of attention. Further, individual differences in exploratory behaviour were observed on the free-viewing task: all metrics were highly correlated across working memory load blocks. These findings suggest a need for further investigation of individual differences in eye-movement behaviour; potential factors associated with these individual differences, including working memory capacity and persistence versus flexibility orientations, are discussed.

**Supplementary Information:**

The online version contains supplementary material available at 10.3758/s13414-023-02741-1.

## Introduction

When navigating the visual world, most scenes we encounter in daily life contain too much information to be processed in totality. Visual attention plays a vital role in managing this mismatch between cognitive demands and capabilities, selectively triaging the processing of salient information at the expense of irrelevant input (Goodhew, 2020a). An important aspect of this selective processing is *spatial attention*, which involves the allocation of attention to specific subregions of the visual field. Spatial attention is a process that is vital to the daily functioning of most people, being heavily implicated in tasks such as driving (Wolfe et al., 2020) and reading (Franceschini et al., 2012). There are two major ways in which spatial attention is deployed across visual scenes: (a) the spatial extent of the attended region around the point of attentional fixation (often termed *attentional breadth*) can be changed (Feldmann-Wustefeld & Awh, 2019; Muller et al., 2003; Sasaki et al., 2001), and (b) the spatial location of this fixation can be *shifted* around the visual field (Kustov & Robinson, 1996; Sauseng et al., 2005; Yantis et al., 2002). For example, one might adopt a narrower breadth of attention to read the words on this page, but adopt a broader breadth of attention to judge the length of this paragraph. Conversely, one must move a narrow focus of attention to new locations to continue reading new text.

While both kinds of spatial attentional deployment can occur in response to task-specific demands, evidence also indicates that individuals have distinct preferences or tendencies to deploy their attention in different ways in the absence of such demands. For attentional breadth, several studies have demonstrated a dissociation between an individual’s *ability* to control the breadth of attention to perform a task as instructed, versus their *preference* for adoption of a broader or narrower attentional breadth in the absence of other task demands. Caparos et al. (2013) found evidence for this dissociation in a cross-cultural context comparing British and Himba observers, showing that although Himba participants demonstrated a *preference* for a narrower attentional breadth when the task did not require or benefit from either a narrow or a broad breadth of attention, they also demonstrated superior *ability* to adopt both narrow and broad breadths of attention in tasks where these respective breadths were required for task performance. Similarly, Koldewyn et al. (2013) found that while autistic children demonstrated a preference for a narrow attentional breadth relative to their neurotypical peers in a subjective matching task, these groups did not differ in their ability to adopt a broader breadth of attention when the task required it. While some studies have contrasted ability and preference in this way to highlight their dissociation, many others have focused solely on either ability (Chong & Treisman, 2005; Goodhew et al., 2017, 2016; Notebaert et al., 2011; Pringle et al., 2001) or preference (Elahipanah et al., 2011; Lawrence et al., 2018; McKone et al., 2010; Rowe et al., 2007) measures of attentional breadth. The defining difference between these two approaches is whether task performance requires or benefits from the adoption of a particular attentional breadth. If so, then ability is measured; if not, then preference is measured. For example, tasks that require participants to identify a target at a fixed level of detail (local vs. global level) of a hierarchical stimulus measure ability, whereas tasks where the target is equally likely to appear at either level of detail gauge preference.

Applying this ability versus preference distinction to the measurement of attentional shifts, many laboratory tasks requiring shifts of attention lead individuals to adopt similar patterns of eye movements to one another (e.g., serial shifts of attention around a memory array to encode items). These would be considered ability measures on the basis that a particular pattern of attentional shifts is the optimal strategy for task completion. However, other task designs that do not clearly impose an optimal pattern of information acquisition can reveal individual *preferences* for attentional shifting behaviour. These preferences can be thought of as distinct ways of *exploring* a visual scene in the absence of a specific target that is searched for. Eye tracking provides a particularly useful way to measure such exploratory spatial deployment of attention. The metrics derived from eye tracking can be categorised into two types: firstly, *exploratory breadth*: the spatial extent of the region over which saccades are being made, and secondly, *exploratory dynamics*, reflecting *how* this region is explored. Some key metrics of exploratory dynamics derived from eye-tracking data include fixation duration (how long fixations are held during image viewing), saccade amplitude (the length of saccades made during image viewing), and scan-path length (the summed length of all saccades made when viewing an image). It is known that when completing tasks where there is no clearly optimal pattern of eye movement such as scene memorisation, participants demonstrate individual differences in the eye-movement behaviour that they adopt (Carter & Luke, 2018; Cronin, Hall et al., 2020; Cronin, Peacock et al., 2020; Loh et al., 2022; Luke et al., 2018; Mills et al., 2011). Further, these individual differences in scan patterns are predictive of other cognitive constructs such as working memory capacity (Hayes & Henderson, 2017).

However, while individual behavioural preferences can be observed in the spatial deployment of attention, these deployments are also sensitive to contextual manipulations. One such contextual manipulation that appears to affect spatial deployments of attention is *working memory load* (the amount of information that the working memory system is required to hold at a given time). The relationship between working memory and spatial attention has long been of theoretical interest, as working memory subserves optimal and efficient allocation of attention (Oberauer, 2019). Conversely, higher working memory loads are generally associated with impairments in selective attention, such that processes dependent on attentional control are executed less efficiently (Lavie, 2010; Lavie et al., 2004). This interplay between working memory load and allocation of spatial attention is also of major applied significance, with clear implications for cognitively demanding daily tasks such as driving (Johannsdottir & Herdman, 2010; Ross et al., 2014; Walshe et al., 2019).

Given that higher working memory loads tend to cause difficulties with selective attentional control, how might this effect manifest for spatial deployments of attention? For attentional breadth, humans are typically thought to process scenes with a broad attentional breadth to encode the gist of a scene before changing to a narrow one to encode the details (seeing the ‘forest’ before the ‘trees’; Bar et al., 2006; Furtak et al., 2022; Navon, 1977), and people generally take longer to contract their attention than to expand it (Goodhew & Plummer, 2019). This suggests that a broader attentional breadth may be a general ‘default’ that is less demanding to instantiate than a narrow attentional breadth. Consistent with this, attentional breadth is generally found to *broaden* or ‘de-focus’ under higher working memory load (Ahmed & de Fockert, 2012a, b; Caparos & Linnell, 2010; Linnell & Caparos, 2011). This is consistent with a failure of selective attentional control, such that higher working memory load impairs the ability to focus attentional resources over a small spatial area. However, it is important to note that a broadening of attentional breadth under high working memory load is not always observed (Hoar & Linnell, 2013; Li et al., 2019; Yao et al., 2020), and more evidence is therefore needed to assess this hypothesis.

If attentional breadth does broaden under higher load due to a failure of selective control, what might this impairment of attentional control under higher working memory load look like if also present for exploratory behaviour? Consistent with a decrease in efficiency of processes dependent on attentional control such as saccade planning and execution (Nuthmann et al., 2010; Trukenbrod & Engbert, 2014; Walshe & Nuthmann, 2021), operating under a higher working memory load may decrease the amplitude of saccades and hence shorten scan paths, while also increasing fixation durations. In turn, this decreases exploration of areas of the stimulus that are further out from the centre, *contracting* exploratory breadth. Indeed, this pattern of performance is exactly what was observed in one recent study (Cronin, Peacock et al., 2020). Even in contexts where performance ability rather than preferential behaviour is being measured, such as visual search tasks performed under increasing working memory loads, a relationship between load and eye-movement behaviour still results. He and McCarley (2010) found that fixation durations increased under higher working memory load in both of two visual search experiments. Similarly, in Peterson et al. (2008), longer fixations were observed under higher working memory load when completing a visual search task, although this effect was only significant for items that were not revisited when scanning a scene.

However, prior research investigating the impact of working memory load on spatial deployments of attention has most often assessed the difficulty of *maintaining* representations of information in working memory. Conversely, one aspect of working memory load that remains largely untested is how dynamically *updating* or *manipulating* information held in working memory affects exploratory breadth. While maintenance and updating are complementary functions of working memory, they are subserved by different neural mechanisms (Nyberg & Eriksson, 2015), and the process of updating working memory has reliably been modelled as a process separate from maintenance (Bledowski et al., 2009; Ecker et al., 2010). Furthermore, maintenance and updating of working memory can have differential effects on attentional processes: Boal et al. (2018) found that attentional biases to threat information in social anxiety were not affected by a load manipulation that required participants to maintain information in working memory, but Delchau et al. (2020) were able to eliminate the same bias toward threat in social anxiety by using a load manipulation that required updating/manipulation of information in working memory. Given the practical relevance of updating (as opposed to maintaining) information in working memory for a task with as many spatiotemporally dynamic inputs as driving, assessing the relationship between working memory loads related to updating representations and spatial deployment of attention is an important avenue of research.

In summary, the focus of this study was to compare how two core measures of spatial attentional deployment (exploratory eye movements and attentional breadth preference) were affected by an updating-based working memory load within the same group of participants. Therefore, in the present study, participants completed a naturalistic scene-viewing task in which their exploratory eye-movement behaviour was measured, as well as a task designed to gauge their attentional breadth preference. They completed both tasks under a lower and a higher updating working memory load to elucidate the impact of an updating working memory load on exploratory eye movements versus attentional breadth preference.

## Methods

### Participants

Sample size was determined based on a priori power analysis conducted in G*Power (Faul et al., 2007). The selected method of analysis was a 2 × 2 within-subjects repeated-measures ANOVA, such that there was 95% power to detect an effect size of 0.25. Consequently, the appropriate sample size was determined to be 36 participants; an extra 20% allowance for exclusions and technical issues was factored into this sample size, so 43 participants were ultimately recruited. Eligibility criteria for participation were an age of from 18 to 40 years inclusive, and normal or corrected-to-normal vision.

For these 43 participants, mean age was 21.56 years (SD = 4.26 years). Sixteen participants were male, 26 participants were female, and one participant was non-binary. Forty-two participants reported being right-handed, and one left-handed. Five participants reported wearing contact lenses, eight participants reported wearing glasses, while the remaining 30 participants had normal vision. For country of birth, 14 participants reported an East Asian country (e.g., China, Japan, Taiwan), seven reported a South East Asian country (e.g., Indonesia, Malaysia, Singapore), three reported a South Asian country (e.g., India, Pakistan, Sri Lanka), 13 reported being born in Australia, while six reported being born in another country (e.g., UK, USA, South Africa). All participants provided written informed consent before participation, and were able to withdraw from participation at any time without penalty. All ethical aspects of the experiment were approved by the Australian National University’s Delegated Science and Medical Human Research Ethics Committee (protocol 2022.119).

### Materials and procedure

Participants were tested individually in a laboratory room, sitting 80 cm from a 24-in. BenQ XL6430T monitor with a refresh rate of 60 Hz. When completing the study, participants used a fixed chinrest to ensure that their heads remained stable during eye tracking. Eye-movement data were recorded using a desktop-mounted Eyelink 1000 Plus eye tracker, which sampled the right eye at 1,000 Hz. All stimuli were presented and responses recorded via MATLAB’s Psychophysics Toolbox (Brainard, 1997).

After informed consent was obtained and demographic information was collected, participants completed four tasks in a randomised order. Tasks designed to measure attentional breadth and exploratory breadth were administered twice (once under a low working memory load, and once under a high working memory load). Participants were given task instructions by the experimenter, and completed a practice block (a small number of sample trials with feedback provided). Participants undertook the backwards-counting working memory load task (see below) while completing the practice, with instructions to try and count backwards by approximately one number every 2 s. If a prescribed performance threshold was not met, participants repeated the practice task until able to pass it, and then progressed to completion of the experimental block. After completing the four tasks, participants were then debriefed about the purpose of the experiment. Experiment sessions took approximately 60 min on average, and participants were compensated with either a cash payment or research participation credit for relevant undergraduate courses.

### Experimental tasks

#### Attentional breadth

**Fig. 1:**
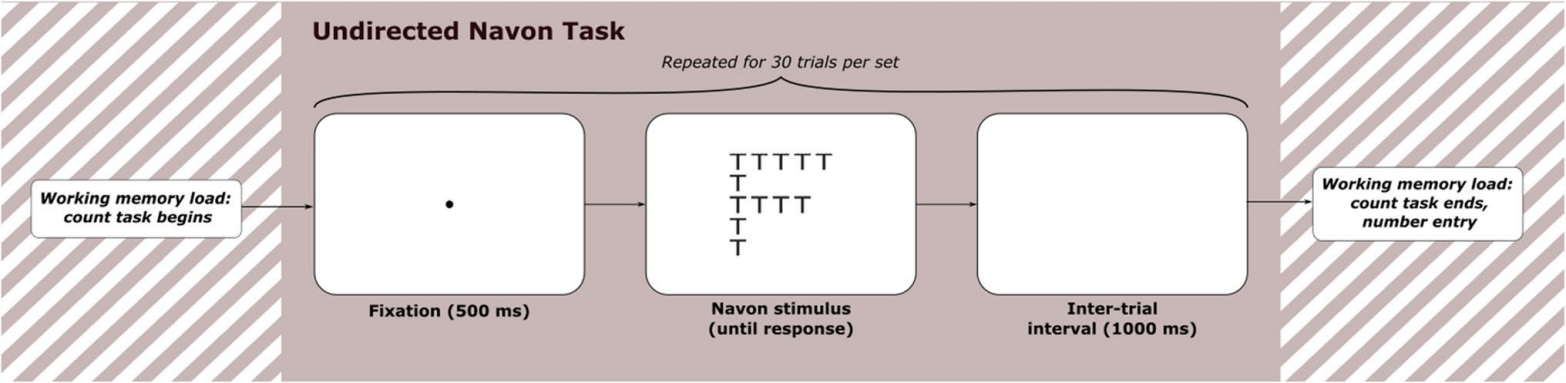
Trial procedure for undirected Navon task. *Note.* There were four sets of trials within a block. For each set, participants continuously completed the working memory load task while undertaking a sequence of 30 consecutive trials. Participants entered their final count number at the end of each trial sequence

To measure attentional breadth, response times to identify the target in Navon figures (Navon, 1977) where the target was featured at the global level were subtracted from response times where the target featured at the local level. To assess whether attentional breadth changed under increasing working memory load, this difference was calculated for each working memory load condition. In the undirected version of the Navon task used here, participants were instructed to identify which of two target letters (T or H) were present in the stimulus shown on each trial as quickly and accurately as possible. Participants entered their responses using the T and H keys on a standard keyboard. Eight Navon stimuli were used, all of which contained one and only one possible target letter; an example is shown in Fig. 1. Using the nomenclature of GLOBALLETTER-localletter, the eight stimuli were: Te, Tf, He, Hf, Et, Ft, Eh, and Fh. Global figures subtended 3.5 × 3.5° of visual angle, while each local letter subtended 0.6 × 0.6°. Navon figures were black, the letters were always uppercase, and figures were presented centrally on a white background.

The trial procedure for our Navon task is shown in Fig. 1. Firstly, a fixation dot was presented in the centre of the screen for 500 ms, then the Navon stimulus appeared on the screen until the participant entered a keyboard response. A 1,000-ms inter-trial interval then occurred before the next trial began, during which time the screen was blank. RTs were recorded from the onset of the Navon figure’s display. Participants completed 120 trials total for each working memory load block, with rest breaks terminated at the participant’s discretion offered after 30, 60, and 90 trials.

This version of the Navon task was *undirected* (Goodhew, 2020), meaning that participants did not receive any instruction to attend to a specific level of the Navon figures being shown, and instead had to identify for themselves which level of the figure was relevant on each trial. While target-local and target-global Navon figures always had an equal chance of appearing, a target would only ever appear at one level of a stimulus. This contrasts with the *directed* version of the Navon task used in previous experiments gauging the effect of working memory load on attentional breadth (Ahmed & de Fockert, 2012b; Li et al., 2019), where participants are directed to attend exclusively to one level of the Navon figures being shown and instructed to identify the target at that level. In the directed version, congruent and incongruent trials are used to gauge the magnitude of interference from the non-target level on target responses. It is thought that the directed Navon task operationalises participants’ capacity to regulate or *control* their attentional breadth to adopt a prescribed attentional breadth for a block of trials, whereas undirected Navon tasks operationalise participants’ tendency to adopt a broad versus narrow attentional breadth when the task does not compel or favour a particular breadth (Caparos et al., 2013; Goodhew, 2021; Koldewyn et al., 2013). For example, if an individual tends to adopt a broad attentional breadth for the undirected Navon task, then they will have to resize their attentional breadth on the local-target trials, which takes time. Therefore, a response-time advantage for the global trials indicates a greater tendency to adopt a broad attentional breadth. Whereas previous work examining the effect of working memory load has used directed Navon tasks and therefore has likely operationalised the control of attentional breadth, here we were interested in how working memory load affects participants’ *tendency* to adopt broad versus narrow breadths of attention. This provides a better analogy to eye-movement behaviour in a free-viewing context: what do individuals do with their spatial attention when a task does not systematically compel them to do one thing or another?

Individuals typically respond quicker on global relative to local trials in an undirected Navon task (Calcott & Berkman, 2014; Goodhew, 2020b; Goodhew & Plummer, 2019), although such global advantages are moderated by a number of variables including the relative sizing of global and local stimulus elements (Yovel et al., 2001). If participants’ attention broadened under higher working memory load, the response cost for resizing attention to identify a local element of a Navon figure, relative to the global form of a figure, should increase the response time on local trials, thereby enlarging the response-time advantage for the global trials.

#### Exploratory breadth

To gauge whether metrics of exploratory breadth changed under a higher working memory load, we used a free-viewing scene-recall paradigm (Cronin, Hall et al., 2020, Cronin; Peacock et al., 2020; Loh et al., 2022) in the form of a simple memory test in each load condition. Scene-viewing behaviour is known to be sensitive to changes in retrieval-based working memory load even in the absence of clear task demands (Cronin, Hall et al., 2020; Cronin, Peacock et al., 2020). This free-viewing recall task gauges participants’ preferred eye-movement behaviour in the absence of any clear optimal strategies: unlike other tasks involving specific objects to fixate such as visual search or object tracking, memorisation of complex naturalistic scenes for later recall does not impose task demands that lead participants to converge on similar patterns of eye movements (Mills et al., 2011).

**Fig. 2:**
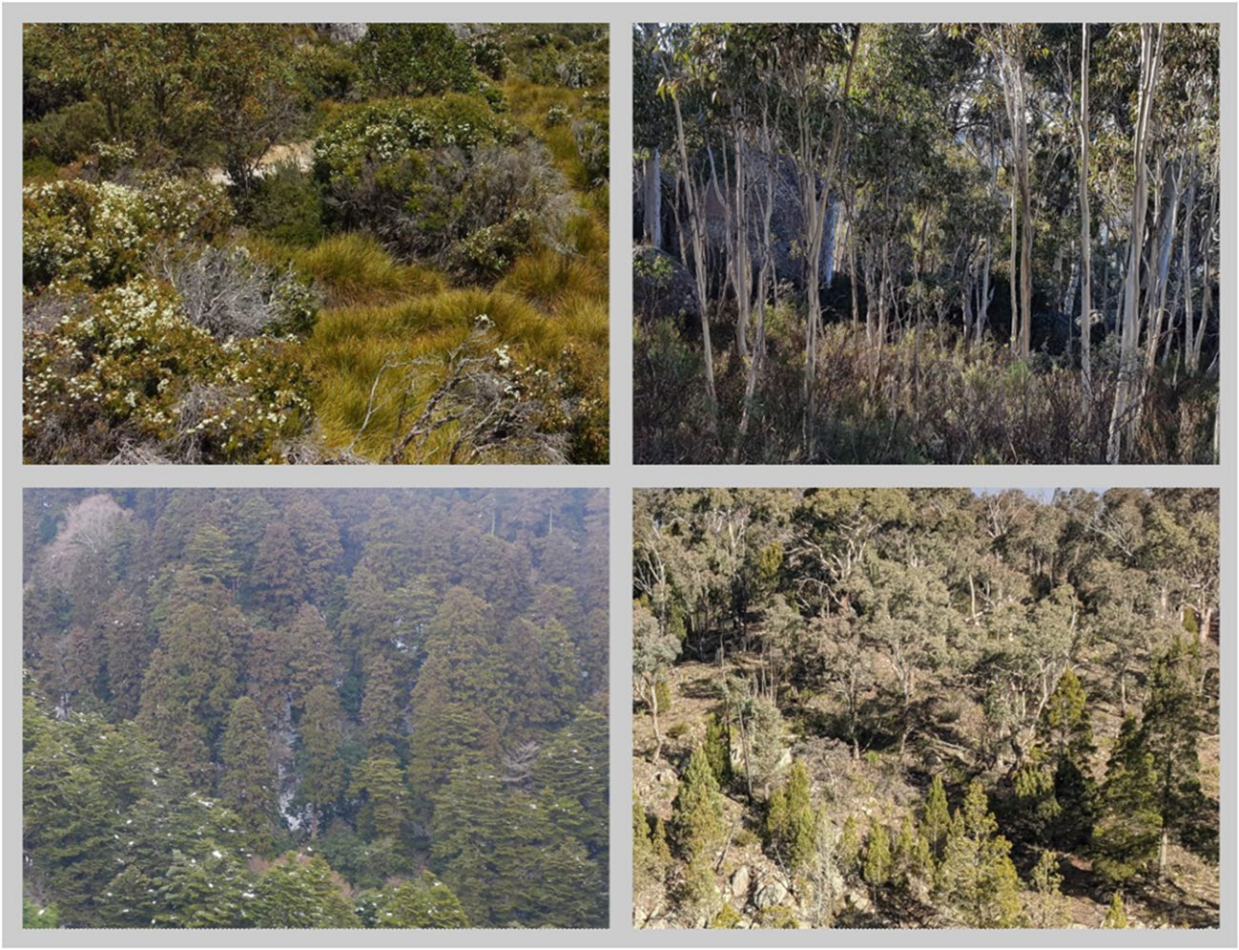
Examples of stimuli used in the scene-recall task

Our design added a memory test to assess recall of the scenes viewed for two reasons: firstly, to reduce demand characteristics that may be associated with participants viewing scenes without any assigned objectives when they know that their eye movements are being tracked, and secondly, to assess whether recall of scenes also varied as a function of working memory load. The set of 96 images used for this study were photographs of natural scenes, without any clearly salient features or compositional structure. Photographs were presented against a grey background (RGB: [172, 147, 147]). Examples of the images used in this task are seen in Fig. 2.

**Fig. 3:**
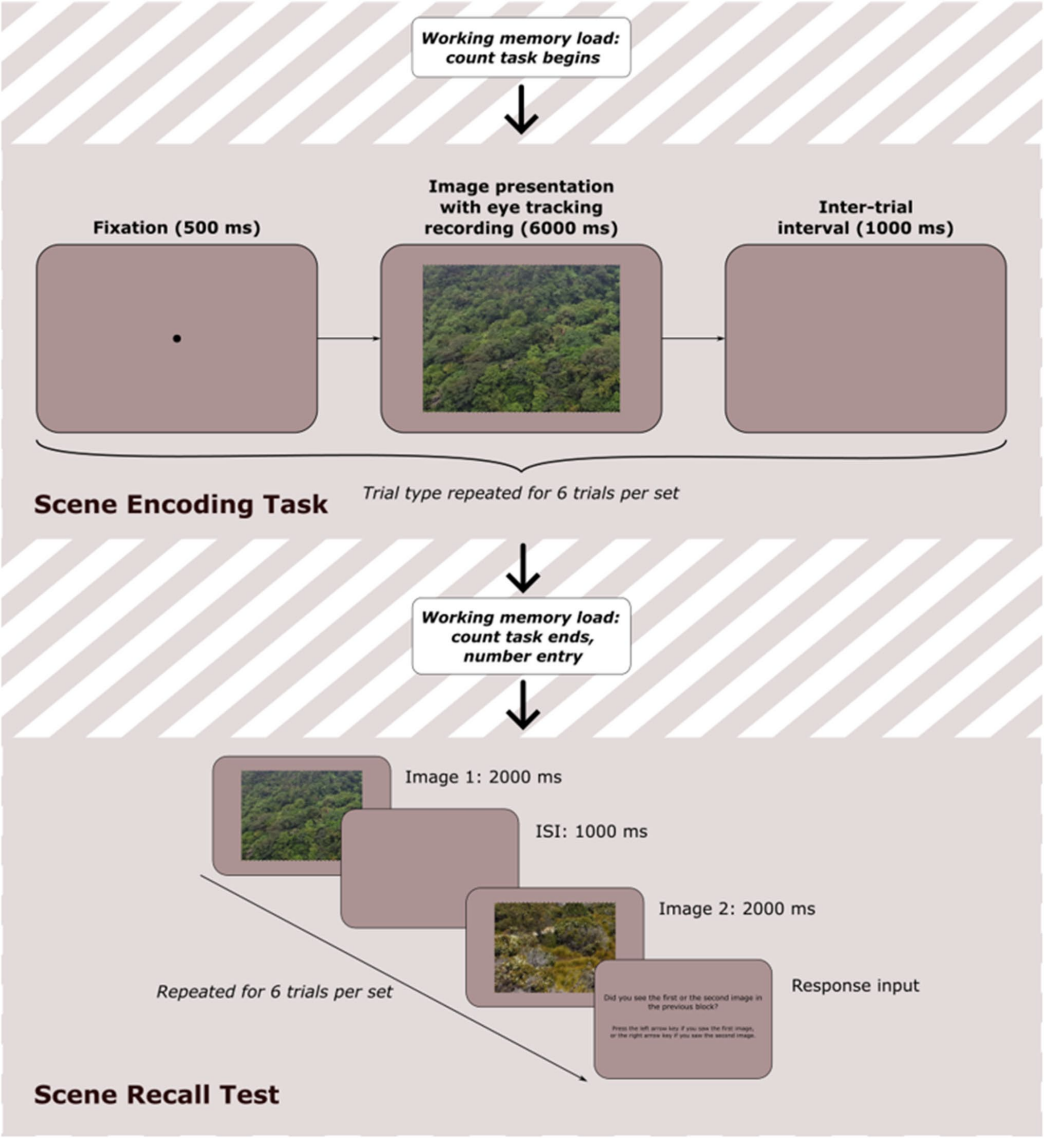
Trial procedure for scene recall task. *Note.* There were four sets of trials within a block. For each set, participants continuously completed the working memory load task while undertaking a sequence of six scene-encoding trials. At the end of each scene-encoding trial sequence, participants entered their final count number

For each working memory load block, participants completed four sets of six scene-viewing and memory-recall trials each, for a total of 24 of each trial type per block. Prior to each scene-viewing set, participants underwent Eyelink’s standard 9-point eye-tracking calibration procedure. As seen in Fig. 3, each scene-viewing trial began with a fixation dot being presented in the centre of the screen for 500 ms, after which time eye-movement data recording began and an image in landscape orientation subtending 22.5 × 17.0° of visual angle was presented for 6,000 ms. After the image presentation terminated, eye-movement data recording ended and a blank screen was shown for a 1,000-ms inter-trial interval.

After six scene-viewing trials, participants completed six memory-recall trials. On each memory-recall trial, two images were shown consecutively for 2,000 ms each, with a 1,000-ms blank screen between: one was an image that had been seen during the prior scene-viewing block, while the other was a different image that had not been seen before. Participants were asked to identify whether they had seen the first or second image in the previous scene-viewing block, pressing the left arrow key on a standard keyboard if they had seen the first image, and the right arrow key if they had seen the second image. Whether the first or second image was the one that they had seen before was randomised, and there was no correspondence between the trial number on which an image was shown in the scene-viewing block, and the trial number on which it appeared in the memory-recall block. There was also no repeat of images between the two working memory load blocks.

We extracted three metrics of interest for each individual participant to investigate the spatiotemporal dynamics of exploratory behaviour:

- Event-level means and standard deviations of fixation duration (i.e., all fixation durations were analysed for each participant without averaging at trial level)
- Event-level means and standard deviations of saccadic amplitude
- Event-level exploratory breadth (Euclidean distance of each fixation from image centre)
- Trial-level scan-path length (sum of all saccadic amplitudes on each trial, representing the distance travelled by all saccades).

If increased working memory load causes a failure of selective control for eye-movement behaviour, it might be expected that mean fixation durations increase while mean saccadic amplitudes decrease, as both patterns of performance would correspond to lower availability of executive resources that can be dedicated to eye-movement planning and execution. Conversely, standard deviations for both metrics may increase as eye movement behaviour becomes more erratic and less systematic. Finally, these behaviours may decrease exploration of areas of the stimulus that are further out from the centre, thus *contracting* exploratory breadth and reducing scan-path length.

#### Working memory load

We wanted to test the impact of a working memory load manipulation that gauged participants’ ability to update or manipulate information held in working memory, rather than simply maintaining representations of information. We also wanted to avoid the presentation of visual information on each trial to manipulate experimental working memory load, because this visual information could potentially influence attentional breadth separate from its load effect (e.g., more widely spaced working memory inducers are known to broaden attentional breadth; Lee & Jeong, 2020). Finally, we also wanted to selectively test for effects of working memory load, rather than generic dual-task effects, and therefore compared the effect of two different loads, rather than the presence of a load to the absence of a load. We therefore elected to use a simple arithmetic task to load working memory, whereby participants counted backwards out loud by ones (low working memory load) or threes (high working memory load) from a randomly generated three-digit number while completing the other tasks that measured the spatial deployment of attention. Mental arithmetic loads the central executive function of working memory (Engelhard et al., 2011; Imbo & Vandierendonck, 2007), while subtraction specifically is also known to load phonological and visuospatial working memory subsystems (Cavdaroglu & Knops, 2016; Lee & Kang, 2002).

At the start of each block of trials for both the Navon and scene-viewing tasks, participants saw a screen with a randomly generated three-digit number above 500. They were instructed to begin counting backwards out loud from this number in increments of either one or three depending on whether the task was being completed under low or high working memory load, respectively. Once counting fluently, participants pressed a key to begin completing the screen-based task simultaneously. Participants were monitored for working memory load task compliance: the experimenter ensured that the counting activity was continuously undertaken for the duration of the screen-based task. After each block of 30 trials was complete for the Navon task, or the six scene-viewing trials were complete for the scene-viewing task, participants saw another screen where they inputted the most recent number they had spoken. Participants did not undertake the backwards counting task while completing the memory-recall component of the scene-viewing task.

Several steps were taken to ensure task compliance, including: (1) having participants practice completing the working memory load task at a pace of approximately one number per 2 s; (2) having the experimenter remain in the room with the participant for the duration of experiment monitoring for counting task compliance, with prompting to continue counting after a silence of more than 5 s; and (3) assessing whether their final count number was consistent with their starting number and count intervals. Participants were able to maintain a reasonable counting pace (i.e., at least one number every 5 s) approximately 91% of the time; see Online Supplementary Materials (OSM) for full details on compliance with the working memory load task.

### Data preparation

Eye-movement data were extracted using Data Viewer for Eyelink and processed in MATLAB. Fixations and saccades were segmented in accordance with Eyelink’s standard algorithm using velocity (30°/s) and acceleration (9500°/s^2^) thresholds. Five participants with track-loss rates of greater than 25% were removed from further analysis and replaced with new participants. Before further analysis, screening of eye-movement data was also applied at trial and event levels to improve data quality. For fixation data, fixations outside the bounds of the presented image, as well as those with extremely short (< 50 ms) or long (> 1,500 ms) durations, were excluded, resulting in a loss of 6.9% of fixation datapoints. Saccades of greater than 28.2° (i.e., the diagonal dimension of the presented image), as well as those with zero and null values, were removed, resulting in a loss of 0.8% of saccade datapoints. Finally, scan paths were calculated by summing the total Euclidean distance covered by saccades on each trial; trials with missing saccade data were removed, resulting in a loss of 1.1% of trial scan-path datapoints.

## Results

### Overview

Raw data for this study are available via the Open Science Framework at this link: https://osf.io/uszd6/. This *Results* section is divided into the following subsections: data screening and participant exclusions are summarised, and then the effects of the working memory load manipulation are considered for the Navon and scene-recall tasks.

### Data screening and participant exclusions

Sample size was calculated at *n* = 36 (see *Methods* section), so 43 participants took part in the study (allowing an additional 20% for participant exclusions). After two participant exclusions following screening for outliers, the final sample size was *n* = 41, which exceeded the required power for intended statistical analyses.

For the Navon task, the main dependent variable of interest was response time (RT) on correct-response trials. RTs were first subjected to trial-level analysis: trials were removed from further analysis if participants responded too quickly (< 100 ms) or too slowly (> 2.5 SDs above participant’s mean RT; Goodhew et al., 2020). This exclusion was designed to remove trials where the participant was not making a genuine attempt to complete the task as instructed; extremely quick responses are likely pre-emptive, while extremely slow responses likely reflect task disengagement. One participant had more than 10% of trials excluded according to these criteria, and all of this participant’s data were excluded from further analysis. For the remaining participants following screening, the percentage of trials excluded was low (*M* = 3.0%, *SD* = 1.3%). A minimum criterion of 80% accuracy in all conditions was applied to ensure compliance with task instructions; no participants were excluded on this basis. Finally, univariate outlier screening was performed using a z-score criterion (± 3.29, p < .001) for RT (Tabachnick & Fidell, 2013). One additional participant’s data were excluded on this basis.

For the image-viewing task, event and trial-level screening were applied as described in the *Data preparation* subsection of the *Methods* section. All participants performed at or above 66.67% accuracy for the memory probe task, well above chance level (50%), so no exclusions were performed on this basis. The same z-score criterion used for Navon outlier screening was applied to individual participants’ screened means for fixation duration, saccade amplitude, exploratory breadth (Euclidean distance of fixations from centre), and scan-path length; no participants were excluded on this basis.

In total, two participants’ data were excluded from further analysis, for a total sample of *n* = 41 in the final analysis. Results from all primary analyses remained identical with and without exclusions applied (see OSM).

### Navon task performance

**Fig. 4:**
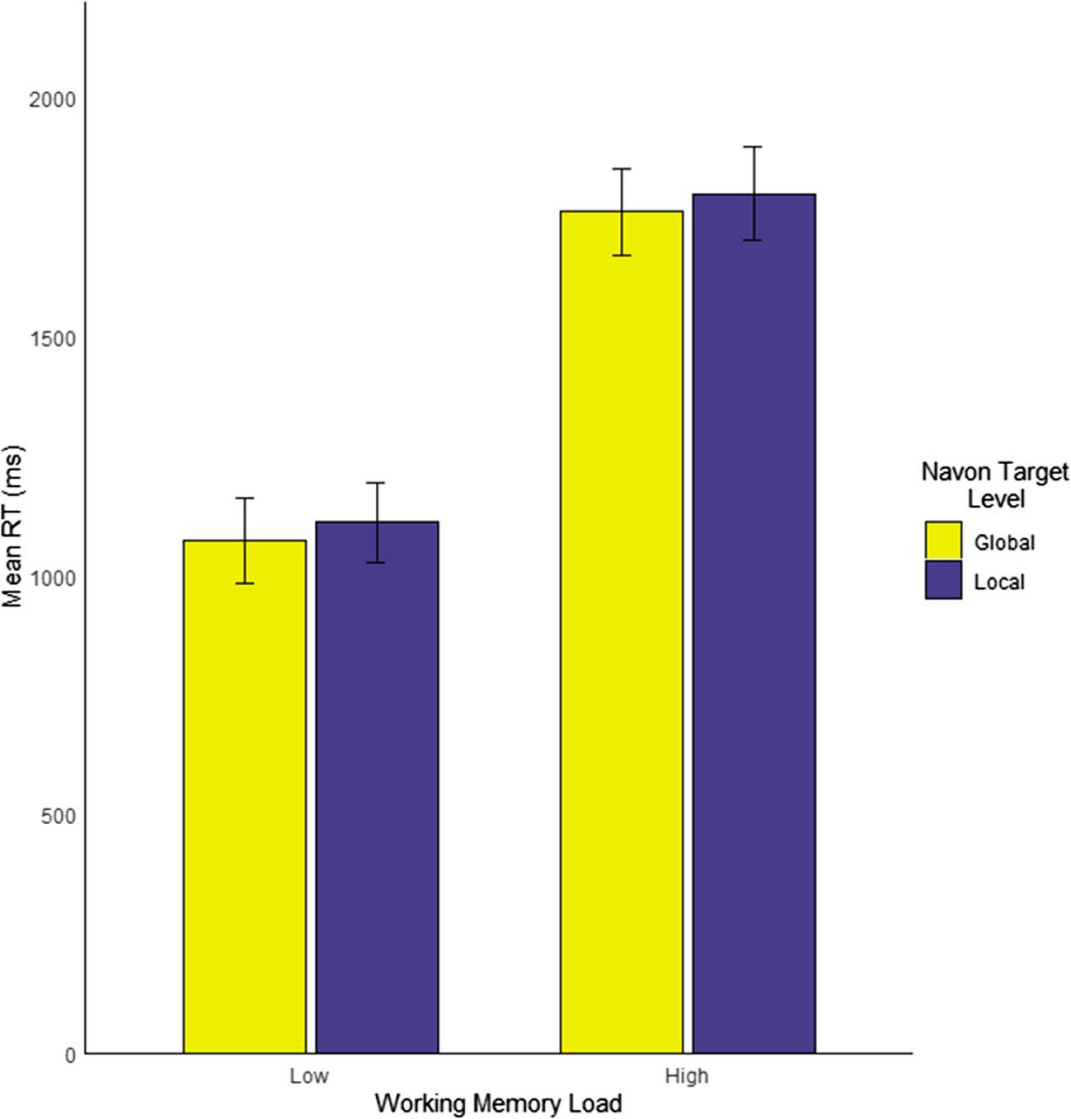
Undirected Navon task response times (RTs) by working memory load and stimulus target level. *Note.* 95% confidence intervals calculated for repeated-measures ANOVA following the procedure in Cousineau (2005)

Descriptive statistics for Navon task performance are reported in Table 1. Mean accuracy for all conditions of the Navon task under both working memory loads exceeded 98% (see Table 1), so analysis focused primarily on RT measures. A 2 (working memory load: low vs. high) × 2 (Navon target level: local vs. global) repeated-measures ANOVA was performed on RTs from correct trials only. There was a significant main effect of working memory load, such that RTs were significantly faster under low load (*M* = 1,091.6 ms, *SD* = 396.6 ms) than under high load (*M* = 1,780.0 ms, *SD* = 591.9 ms); *F*(1, 40) = 62.46, *p* < .001, η^2^_p_ = .610. This means that the working memory load demonstrably impacted participants’ performance. There was no main effect of target level, such that participants did not significantly differ in their responses to target-global trials (*M* = 1,416.9 ms, *SD* = 614.6 ms) versus target-local ones (*M* = 1,454.8 ms, *SD* = 607.5 ms); *F*(1, 40) = 3.20, *p* = .081, η^2^_p_ = .074. This indicates that neither the global nor the local targets were on average more salient to participants as a whole, a common finding with stimuli such as these (Goodhew & Plummer, 2019; Yovel et al., 2001), in contrast to very dense Navon stimuli that can induce a global precedence effect. Finally, there was no interaction between working memory load and target level (*F*(1, 40) < 0.001, *p* = .979, η^2^_p_ < .001). The absence of an interaction suggests that the effect of working memory load did not differ for the global and local targets (Fig. 4).

**Table 1:** Descriptive statistics for Navon task performance

Variable	Low working memory load		High working memory load	
	*M*	*SD*	*M*	*SD*
Accuracy				
Global accuracy (%)	98.6	1.5	99.0	98.9
Local accuracy (%)	98.6	2.0	1.4	2.0
Response time (RT)				
Global RT (ms)	1072.9	389.5	1760.8	608.9
Local RT (ms)	1110.3	407.6	1799.3	581.3

### Eye-movement behaviour: Image-viewing task

To assess the impact of working memory load for the image viewing task, we first considered the impact of load upon memory recall performance, then undertook an in-depth analysis of the impact of load upon four eye-movement behaviours: fixation duration, saccadic amplitude, exploratory breadth (Euclidean distance of fixations from image centre), and scan-path length. Finally, we considered the potential role of individual differences in behaviour on the image-viewing task.

#### Memory test performance

To test the effect of the working memory load manipulation on participants’ memory for the pictures presented in the image-viewing task, memory probe scores for the lower-load versus higher-load conditions were compared with a Wilcoxon signed-rank test (a non-parametric test was used as memory probe scores were not normally distributed). Results indicated that memory accuracy was significantly poorer under higher working memory load (*M* = 85.1%, *SD* = 8.2%) compared to lower working memory load (*M* = 91.8%, *SD* = 7.8%); *z* = -3.96, *p* < .001, *r*_*rb*_^1^ = .790 (95% CI: [.591, .898]). This suggests that our working memory load manipulation was effective even for performance on a relatively minimal delayed recall task, where participants had a full 6 s to passively view and memorise images for identification a short time later.

#### Eye-movement behaviour: Mean and standard deviation analysis

**Fig. 5:**
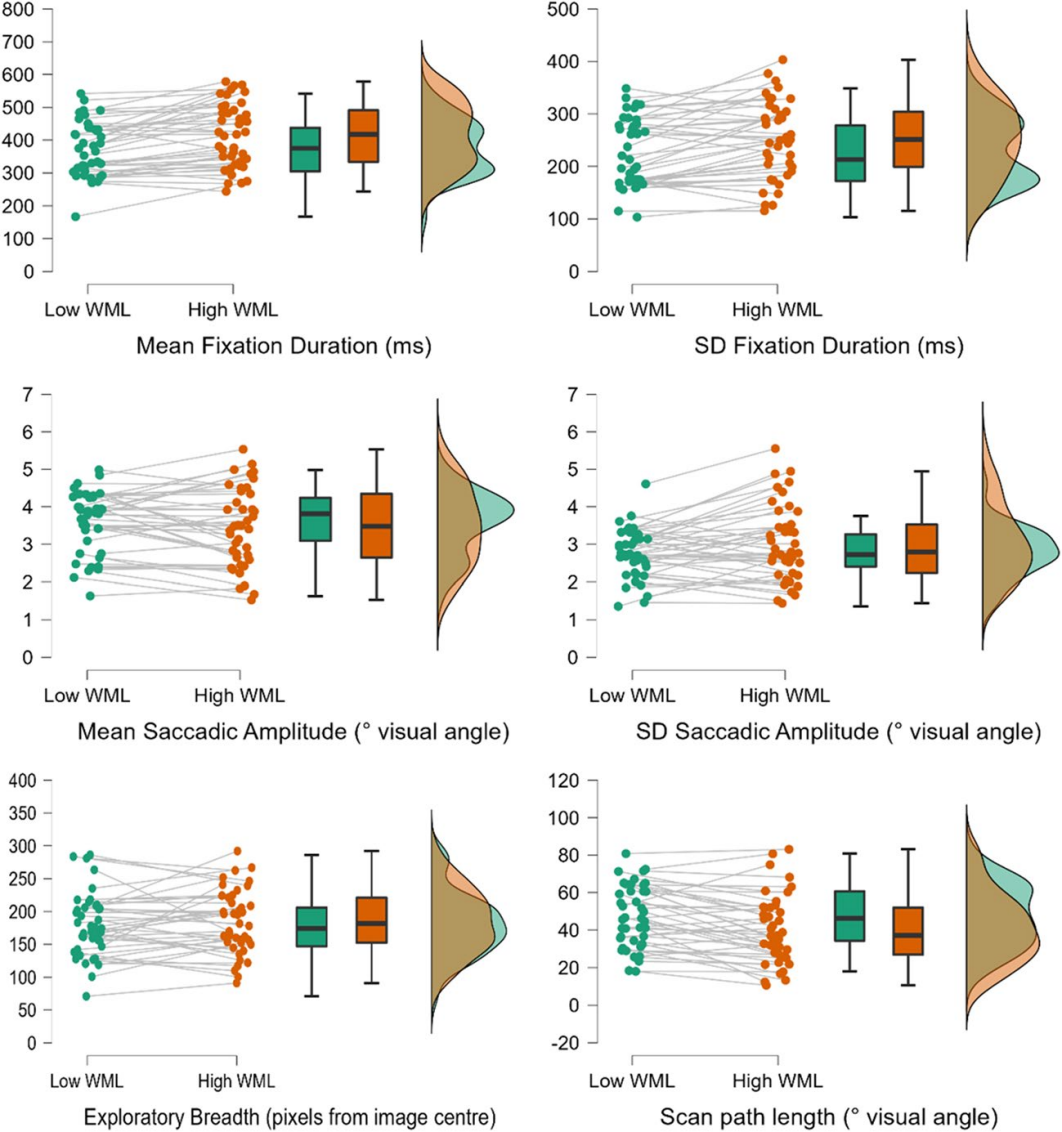
Visualisations for eye-movement data. *Note.* Performance slopes across working memory loads are shown for each participant, as well as boxplots and distributions for sample performance under each working memory load. Performance in the low working memory load condition is shown in green, while performance in the high working memory load condition is shown in orange

The next series of comparisons investigated the impact of working memory load upon the four types of measures for trial-level behaviour on the eye-tracking task. The first three of these measures were the means and standard deviations of (1) *fixation duration* and (2) *saccadic amplitude*, as well as (3) *exploratory breadth* (the mean Euclidean distance of each fixation from image centre), each calculated from all individual datapoints for each participant that remained after event and trial-level screening (see *Methods* section for more information). The final measure, (4) *scan-path length*, was calculated from the total distance in pixels covered by saccades for each trial completed by each participant. Standard deviations as well as means were considered for fixation duration and saccade amplitude to indicate if the *variability* of eye-movement behaviour changed between working memory load conditions.﻿ Visualisations of these comparions are shown in Fig. 5. Initial analyses for each metric are performed using both frequentist and Bayesian t-tests^2^ using the default priors in JASP Version 0.17.2 (2023); Bayes factors are interpreted per the guidelines in Andraszewicz et al. (2014). This indicative analysis was then complemented by fitting ex-Gaussian distributions to each participant’s fixation data.

##### Fixation duration

Comparison of mean fixation durations across working memory load conditions indicated that participants held fixations for significantly longer under high load (*M* = 415.4 ms, *SD* = 97.3 ms) than under low load (*M* = 374.7 ms, *SD* = 83.5 ms); *t*(40) = -4.914, *p* < .001, *d* = -0.767, BF_10_ = 1327.2. The Bayes factor suggests extreme evidence in favour of the alternative hypothesis (i.e., that these conditions do differ significantly). Furthermore, comparison of standard deviations of participants’ fixation duration distributions indicated that fixation durations were significantly more variable under high load (*M* = 251.5 ms, *SD* = 73.4 ms) versus low load (*M* = 225.9 ms, *SD* = 64.8 ms); *t*(40) = -3.747, *p* < .001, *d* = -0.585, BF_10_ = 50.5. Here, the Bayes factor suggests very strong evidence that standard deviations of fixation duration differ across working memory load.

##### Saccadic amplitude

Comparison of mean saccadic amplitudes across working memory load conditions did not indicate a significant difference in performance between the high-load (*M* = 3.45°, *SD* = 1.04°) and low-load (*M* = 3.58°, *SD* = 0.81°) conditions; *t*(40) = -1.254, *p* = .217, *d* = 0.196, BF_10_ = 0.35. The Bayes factor indicates anecdotal support for the null hypothesis (i.e., no difference between saccadic amplitude across working memory load conditions). Conversely, the standard deviation of participants’ saccadic amplitude distributions was significantly larger under high working memory load (*M* = 3.04°, *SD* = 1.02°) than under low load (*M* = 2.76°, *SD* = 0.66°); *t*(40) = -2.458, *p* = .018, *d* = -0.384, BF_10_ = 2.411. Although the frequentist test indicates a significant difference between conditions, the Bayes factor indicates that the strength of evidence in favour of this difference is only anecdotal.

##### Exploratory breadth

Comparison of mean exploratory breadths across working memory load conditions did not indicate a significant difference between the high-load (*M* = 179.2 pixels, *SD* = 48.5 pixels) and low-load (*M* = 184.2 pixels, *SD* = 48.8 pixels) conditions; *t*(40) = 0.837, *p* = .408, *d* = 0.131, BF_10_ = 0.234. The Bayes factor indicates substantial evidence for the null hypothesis in this comparison.

##### Scan-path length

Comparison of scan-path lengths across working memory load conditions indicated that participants’ scan paths were significantly shortened under high load (*M* = 40.5﻿°, *SD* = 18.4﻿°) compared to low load (*M* = 47.5﻿°, *SD* = 16.6﻿°); *t*(40) = 3.894, *p* < .001, *d* = 0.608, BF_10_ = 74.8. The Bayes factor suggests very strong evidence in favour of this difference in scan-path length across working memory load conditions.

##### Summary

Overall, comparison of the means and standard deviations of distributions of participants’ eye-movement metrics indicated that a higher working memory load led to significantly longer and less consistent fixation durations and reduced scan paths. In contrast, evidence about the impact of working memory load upon saccadic amplitude was equivocal: while frequentist testing indicated that mean standard deviation did not change under higher load and standard deviation of saccadic amplitude became significantly larger, Bayesian testing indicated that support for these hypotheses was only anecdotal. Finally, these comparisons indicated that participants’ exploratory breadths did not change under higher working memory load.

This pattern of results appears to indicate a change to sampling strategy under higher working memory load: participants generally held fixations for longer, but when shifting their gaze there was greater variability in the timing of these shifts. Conversely, as indicated by the measure of exploratory breadth, a higher working memory load did not alter the extent to which participants explored the images outwards from the centre of presentation.

#### Fixation duration: Ex-Gaussian analyses

In the overall sample, fixation durations averaged across each participant, as well as the standard deviations of participants’ fixation duration distributions, were normally distributed for both working memory load conditions. However, participants’ *individual* fixation duration distributions displayed significant rightward skew, such that there was a tail of longer fixation durations in each participant’s distribution. This skewness of fixation distributions is consistently observed in eye-movement research, and one response is to fit ex-Gaussian distributions to individual participants’ fixation duration distributions (Guy et al., 2020). An ex-Gaussian distribution is produced by combining a normal and an exponential distribution, such that the normal distribution is given a more heavily weighted rightward tail. Ex-Gaussian distributions can be described using three parameters: *mu* (the mean of the normal part of the distribution, indicating average performance for the majority of datapoints), *sigma* (the standard deviation of the normal part of the distribution, indicating the spread of scores for the majority of datapoints), and *tau* (the exponential decay function, indicating the weighting and spread of the rightward tail of outlier trials). Ex-Gaussian distributions therefore allow assessment of the possibility that the longer mean fixation durations seen in the high working memory load condition were driven by changes to the tail of participants’ distributions (i.e., *tau*), rather than to the bulk of the distribution (i.e., *mu* and *sigma*).

**Fig. 6:**
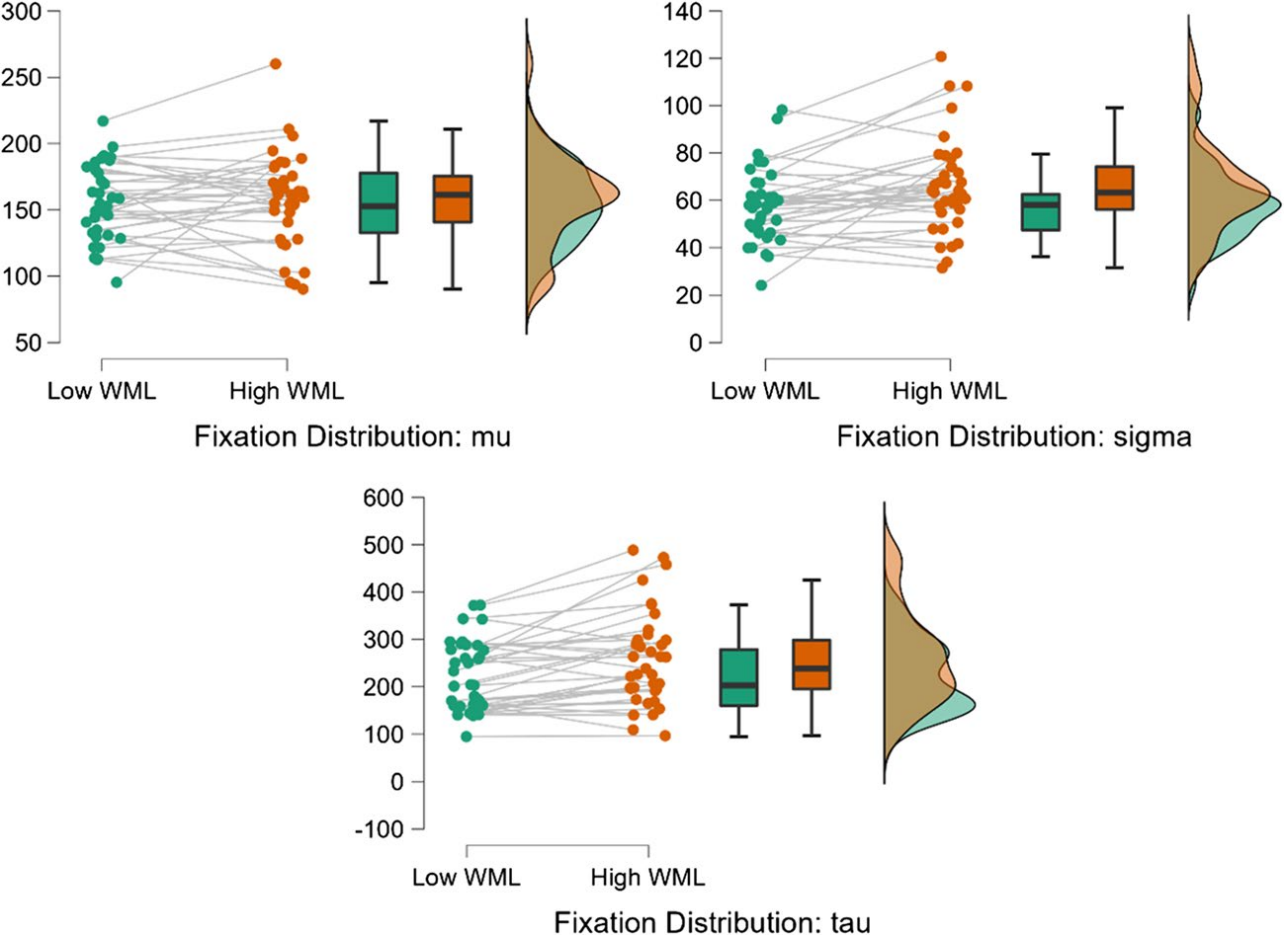
Ex-Gaussian metrics of fixation duration across working memory loads. *Note*. Performance slopes across working memory loads are shown for each participant, as well as boxplots and distributions for sample performance under each working memory load. Performance in the low working memory load condition is shown in green, while performance in the high working memory load condition is shown in orange

Ex-Gaussian distributions were fitted to each participant’s fixation duration data for both working memory load conditions using the *timefit* function of the *retimes* package in R (Massidda, 2013). Four participants for whom the model-fitting process failed to converge were excluded from further analysis of ex-Gaussian parameters (*n* remaining for analysis = 37). Values for *mu*, *sigma*, and *tau* across the two working memory load conditions were then compared with frequentist and Bayesian t-tests^3^ using the default priors in JASP. Values for *mu* did not significantly change between high (*M* = 157.6, *SD* = 35.3) and low (*M* = 154.7, *SD* = 28.1) working memory load conditions; *t*(36) = 0.621, *p* = .539, *d* = 0.102, BF_10_ = 0.212. This Bayes factor indicates substantial evidence in favour of an absence of change to *mu* under differing working memory loads. However, values for *sigma* significantly increased under high (*M* = 66.0, *SD* = 20.1) versus low (*M* = 57.7, *SD* = 15.4) working memory load; *t*(36) = 3.220, *p* = .003, *d* = 0.529, BF_10_ = 12.93. Likewise, values of *tau* also significantly increased under high (*M* = 66.0, *SD* = 20.1) versus low (*M* = 57.7, *SD* = 15.4) working memory load; *t*(36) = 3.809, *p* < .001, *d* = 0.626, BF_10_ = 55.52. These Bayes factors respectively indicate strong and very strong support for the alternative hypothesis (i.e., that *sigma* and *tau* increase significantly under higher working memory load). Figure 6 illustrates the distribution of differences in performance for these parameters.

Overall, examination of ex-Gaussian distributions fitted to participants’ fixation duration data indicates that the increases to mean and standard deviation seen in initial analyses appear to be driven by increases to *tau* and *sigma*, rather than *mu*. In other words, the increasing weight of participants’ distribution tails under higher working memory load, as well as an increase to the spread of the normally distributed portions of their datasets, underlie these increases, while the mean of the normally distributed portion of datasets remains relatively constant.

#### Correlations between measures: Potential individual differences

**Fig. 7:**
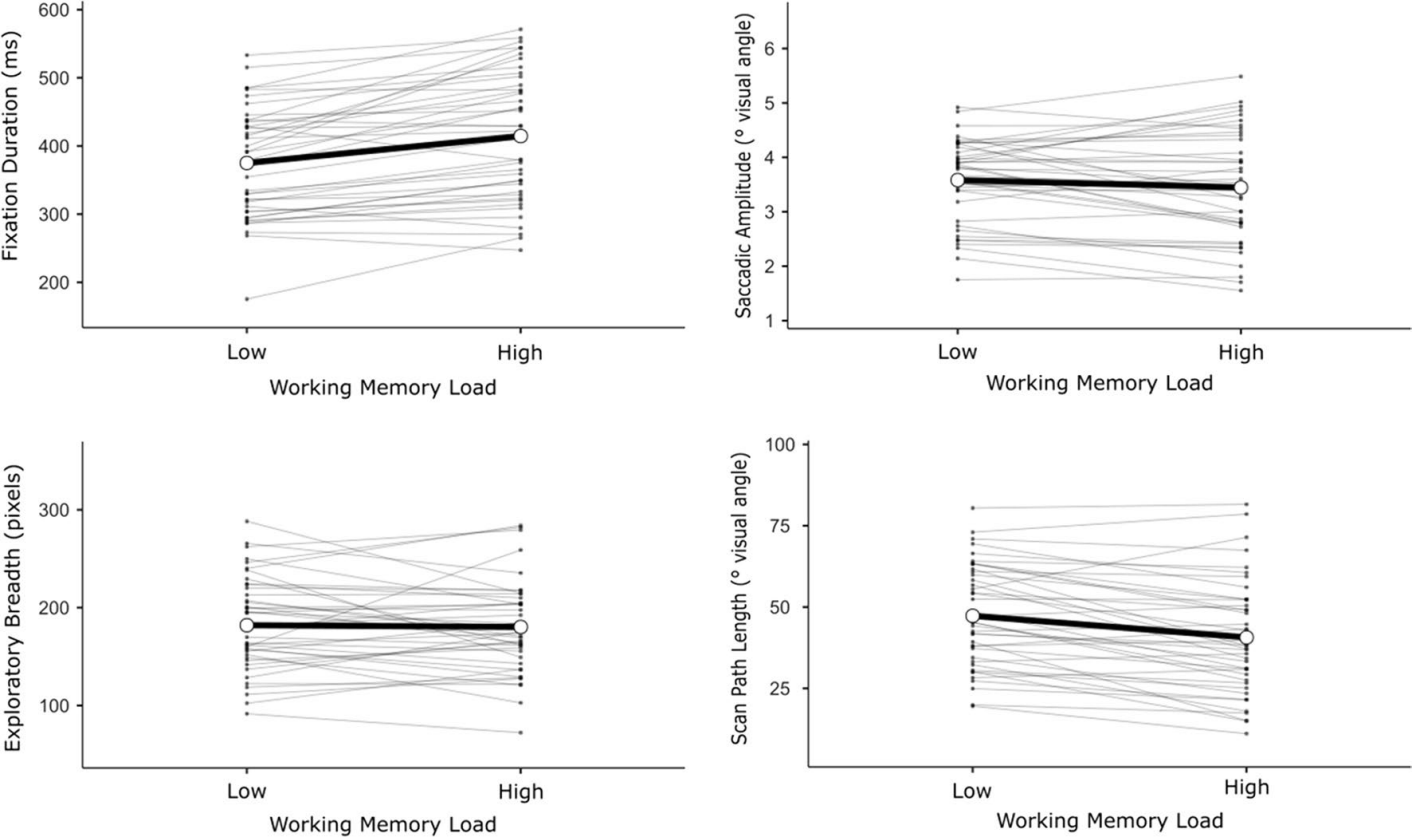
Effects plots for key eye movement metrics across working memory load. *Note*. Thick line is for sample mean effect, thin lines are random effect slopes plotted by participant

To further assess the impact of working memory load upon scene-viewing behaviour, linear mixed-effect model analyses (LMEs) were performed on event-level data for three outcomes: (a) mean fixation durations, (b) mean saccadic amplitudes, (c) exploratory breadth, and trial-level data for one further outcome: (d) scan-path length. The aim of this analysis was to confirm whether behaviour on these eye-tracking measures had been affected by variance unique to individual participants or stimuli. Therefore, in each LME working memory load (lower vs. higher) was entered as a fixed effect, while for random effects, intercepts were entered for participants and stimuli, as well as by-participant random slopes for the effect of working memory load. P-values were obtained by likelihood ratio tests of the full model with the effect in question against the model without the effect in question. Analyses were performed using the GAMLj package for jamovi 2.3.16 (Gallucci, 2022), and effect sizes were calculated according to the procedure in Westfall et al. (2014). Figure 7 shows effects plots for each LME, which show slopes for each individual participant.

Firstly, when predicting fixation durations, the model including working memory load provided a better fit for the data than a model without it (χ^2^(1) = 20.6, *p* < .001, *d* = 0.158), confirming that mean fixation durations increased significantly (*b* = 39.5 ms, 95% CI: [22.4, 56.5]) under higher working memory load. Random effects in this model indicated clustering of fixation durations from each individual participant (ICC = .123), but only very weak correlations between fixation durations for trials of each stimulus type (ICC = .003).

Secondly, when predicting saccadic amplitudes, the model including working memory load did not improve model fit relative to the model without it (χ^2^(1) = 0.93, *p* = 0.337, *d* = -0.043), once more indicating no significant change to mean saccadic amplitude under higher working memory load. Random effects in this model indicated some weak clustering of saccadic amplitudes from each individual participant (ICC = .077), but only weak correlations between saccadic amplitudes for trials of each stimulus type (ICC = .011).

Thirdly, when predicting exploratory breadth, the model including working memory load did not improve model fit relative to the model without it (χ^2^(1) = 0.06, *p* = 0.814, *d* = -0.013), again indicating no significant change to exploratory breadth under higher working memory load. Random effects in this model indicated clustering of exploratory breadths from each individual participant (ICC = .125), but only weak correlations between exploratory breadths for trials of each stimulus type (ICC = .022).

Finally, when predicting scan-path length, the model including working memory load provided a better fit for the data than a model without it (χ^2^(1) = 9.23, p = .003, *d* = -0.243), confirming that mean scan-path length decreased significantly (*b* = -6.63﻿°, 95% CI: [-10.9, -2.36]) under higher working memory load. Random effects in this model indicated strong clustering of scan-path lengths from each individual participant (ICC = .428), but only weak correlations between fixation durations for trials of each stimulus type (ICC = .057).

One noteworthy feature of the LMEs for eye-tracking indices was the intra-class correlation coefficients (ICCs) that emerged for participant-intercept random effects. These ICCs indicated that for each individual participant, performances across all trials were correlated on all variables of interest, regardless of any impact of working memory load. Conversely, consistently weak ICCs for stimulus-intercept random effects affords relative confidence that variance in participants’ eye-tracking indices was not primarily driven by differences in features between stimuli.

**Fig. 8:**
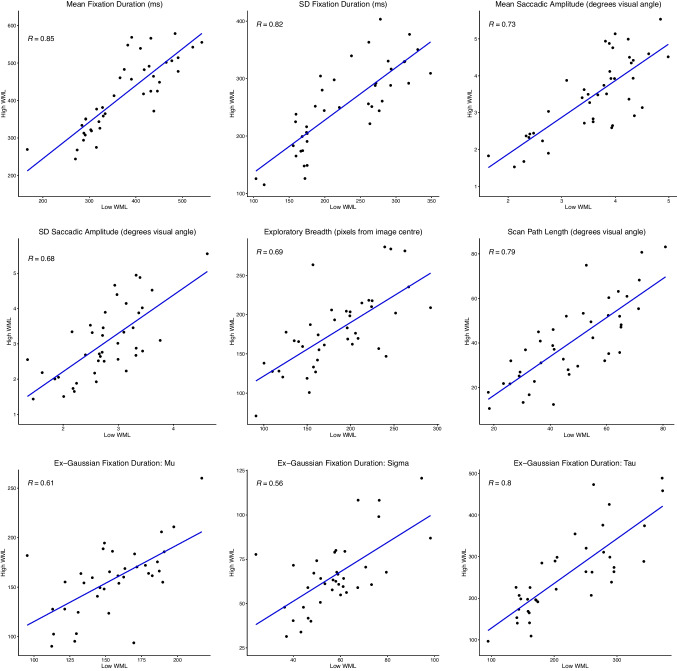
Correlations between performance at low and high working memory loads for eye-movement metrics. *Note.* Spearman correlations reported. All correlations significant at .001 level. Grey shaded area represents 95% confidence interval around the regression line

To illustrate one potential explanation of this observation, if Individual A has a longer fixation duration and Individual B has a shorter fixation duration under low working memory load, then if individuals are consistent within themselves, Individual A should have a longer fixation duration and Individual B a shorter fixation duration under high working memory load as well. If this is true for most individuals in the sample, then this would be revealed via a correlation between an index measured under low load and the same index under high load. Therefore, while the experiment was powered for group-level rather than individual-level analyses, an exploratory analysis was performed where the correlations between eye-tracking indices at each level of working memory load are reported in Fig. 8. These correlations show high degrees of correspondence between individuals’ eye-movement behaviour in the two working memory load conditions, even though they were engaged in different tasks.

## Discussion

The spatial deployment of attention is a cognitive process with critical implications for real-world functioning. However, relatively little is known about how working memory load affects the spatial deployment of attention. This is especially true for the updating and manipulation of representations held in working memory, which clearly parallel the demands imposed by common tasks in daily life such as driving. The core aim of the present study was therefore to assess the impact of a task that required the updating of working memory load representations upon two measures of the spatial deployment of attention: attentional breadth, and exploratory eye-movement behaviours. Attentional breadth was gauged through an undirected Navon task, by comparing RTs for responses on trials where the target was present at the local versus the global level of the figure. Exploratory behaviour was assessed through several metrics based on eye-movement data in a free-viewing paradigm: exploratory breadth was assessed by examining mean distance of fixations from image centre, while exploratory dynamics were assessed by examining fixation durations, saccadic amplitudes, and scan-path length.

In the present study, attentional breadth and exploratory breadth were not affected by increasing working memory load: contrary to tentative initial predictions, attentional breadth did not widen under higher load based on Navon task performance, and the key spatial measure of exploratory breadth (mean distance of fixations from image centre) did not contract under higher load. However, temporally dynamic aspects of eye movements were significantly affected by increasing working memory load: mean fixation durations increased while standard deviation for these durations widened, and mean scan-path lengths shortened. Ex-Gaussian analysis of fixation durations indicated that the impact of working memory load manifested as a more heavily weighted tail of the distribution of fixations, as well as a broader spread of the normally distributed portion of fixations. Conversely, the mean of the normally distributed portion of fixations did not significantly change across working memory loads. Overall, this indicates that while the spatial extent of image exploration did not contract with increasing load, the temporal patterns of exploring similar spatial areas did: fixation durations became less uniformly distributed, which may be linked to the shortening of scan paths under higher load. Furthermore, strong correlations were observed across working memory load conditions for *all* eye-movement metrics, suggesting a role of individual differences in the tendency to deploy spatial attention via eye movements.

The following sections consider the implications of these results. The observed effects of working memory load on exploratory eye-movement behaviours are discussed first, highlighting a potential role for stable individual differences in these behaviours. Two possible reasons why working memory load did not appear to impact attentional breadth in this study will then be discussed in light of prior evidence. Specifically, evidence for a genuine absence of effect from working memory load on attentional breadth will be assessed, and then possible methodological issues that may have affected the ability to observe an effect will be evaluated. Finally, the conclusions that can be drawn about the relative effect of an updating working memory load on exploratory eye movement behaviour versus attentional breadth will be discussed.

### Exploratory eye movements: Experimental effects of load

Using a manipulation that required participants to dynamically *update*, rather than simply store and retrieve the contents of working memory, this study did not observe any changes to preferential exploratory *breadth* (the spatial distribution of exploratory eye movements) under changing load. However, preferential exploratory *dynamics* were clearly affected by this type of load. As load increased, fixations were held longer on average, but duration also became more erratic. Both of these findings can be linked to a changing ex-Gaussian distribution of fixations under higher load, such that the spread of the normal portion of the distribution was wider, and a higher number of exceptionally long fixations caused the tail of participants’ distributions to become more heavily weighted. While frequentist testing indicated that the mean distance of saccadic amplitudes did not change, but that these amplitudes also became less consistent in length under higher load, Bayesian testing indicated that support for these comparisons was only anecdotal, so these findings are not interpreted further here due to the equivocality of this evidence. Finally, mean scan-path length decreased with increasing working memory load, consistent with the less uniform patterns of eye-movement behaviour observed for fixation durations and saccadic amplitudes. Overall, this pattern of results tends to suggest that while the *spatial* extent of exploratory breadth is largely preserved under a higher updating working memory load, the *temporal* aspects of scene viewing change as scan paths shorten. Given that fewer processing resources would be available as load increases, it appears this diminished stock of resources favours the spatial aspects at the expense of the temporal ones – overall, eye movements cover the same spatial locations as they would under a lower load, but this process relies on sampling from fewer fixations as reflected by shorter scan paths.

Why might fixation durations and saccadic amplitudes be affected by changes to working memory load? Regarding fixation durations, eye movements have been described using a mixed-control account: some eye movements are subject to *direct control*, such that processing of information obtained from a specific fixation occurs fast enough to influence the timing of subsequent saccades (and thus the length of the present fixation). Conversely, others are subject to *indirect control*, such that fixation duration is relatively invariant to the properties of the fixated stimulus (Henderson & Pierce, 2008; Nuthmann, 2017; Walshe & Nuthmann, 2021). A unifying characteristic of eye movements subject to direct control is that they are linked to circumstances where acquisition of information is more challenging: when a stimulus percept is degraded (Glaholt et al., 2014; Henderson et al., 2013) or an unfamiliar or unexpected word is encountered when reading (Kliegl et al., 2006), fixation durations increase. Indeed, some accounts of fixation duration in scene viewing (Nuthmann et al., 2010; Trukenbrod & Engbert, 2014) associate ‘difficulties’ in visual and cognitive processing at a specific fixation with slower saccade initiation, or even cancellation of planned saccades. Furthermore, Walshe and Nuthmann (2021) propose an encoding modulation that operates when such difficulties in stimulus processing are encountered, which increases the proportion of long fixations concentrated at the rightward tail of the distribution of fixation durations.

It seems reasonable to assume that operating under a higher working memory load, during which time less central executive processing capacity is available for other tasks, would constitute a circumstance in which such ‘difficulties’ are encountered. In support of this interpretation, memory recall performance for viewed scenes was poorer under higher working memory load in our study. Although it is unclear whether this is attributable to difficulties in extracting visual information in fixated locations, or in encoding this information to short-term memory, recall was clearly impaired under higher load. This explanation is also compatible with converging evidence from Luke et al. (2018), who show that individuals with higher working memory capacity (and thus comparatively fewer processing resource constraints under the same conditions as individuals with low working memory capacity) tend to make fewer very long fixations in scene-viewing paradigms, as well as Meghanathan et al. (2015), who report that fixation duration is an effective index of working memory load in free-viewing paradigms. Consequently, one possible explanation for longer fixation durations under higher working memory load is that processing constraints increase task difficulty and predispose individuals to eye movements subject to direct control, which in turn leads to longer fixations under higher load. Additionally, the wider standard deviation of these fixations under higher load seen using Gaussian distributions was accounted for by the changes to the *tau* parameter in ex-Gaussian analysis: the concentration of very long fixations at the rightward end of the distribution increased as encoding modulations of saccadic behaviour were enacted.

While evidence regarding changes to saccadic amplitude under higher working memory load was equivocal in this study, it is still worth commenting on the potential nature of the relationship between saccadic amplitudes and working memory load. Evidence about this relationship is scant: this finding contradicts Cronon, Hall et al., (2020); Cronin, Peacock et al., (2020), who found a significant reduction in saccadic amplitude under a *retrieval* working memory load, but it is consistent with converging individual differences evidence from Loh et al. (2022), who found that performance on a memory *updating* task similar to ours was *not* correlated with mean saccadic amplitude. This difference may be tentatively attributed to the differing demands imposed by retrieval versus updating paradigms used to load working memory, but further research is needed to substantiate this distinction. Similarly, there is very little evidence about the relationship between saccadic amplitude distribution and working memory load. Two key sources of variability in saccadic amplitude have been identified: firstly, uncertainty in target localisation, and secondly, planning and execution of the movement involved in undertaking a saccade (van Beers, 2007). Working memory load may thus induce a broader distribution of saccadic amplitudes by increasing uncertainty of target localisation, and/or impeding planning and execution of saccades, but once more, further research is needed to substantiate this speculation.

Overall, the results of this study read in combination with existing literature suggest that working memory load is likely to interfere with three key temporal aspects of scene viewing: (1) fixations are held longer on average as information extraction from the visual scene becomes more challenging; (2) distributions of fixation durations widen as an increasing proportion of longer fixations skews the distribution rightward; and (3) saccadic amplitudes may become more variable in length, possibly due to movement planning and execution becoming more difficult when limited resources are available to devote to attentional control. Even if some of the theoretical mechanisms by which these changes occur are unclear, the functional *consequences* of these changes for real-world tasks such as driving are of major importance, as they represent a distinct means for engaging with the visual world when operating under higher load. While beyond the scope of the present study, another important future direction will be further investigation of how manipulation of information in working memory impacts eye movements for tasks that do impose a clear optimal strategy for eye movements (e.g., a visual search task in which there are specific items to find).

### Exploratory eye movement behaviour: Individual differences

Another important finding from this study was the strong correlations observed between all metrics of exploratory eye movement behaviour across working memory loads for individual participants. Despite reliability concerns for individual differences in eye movements in tasks such as reading (Rayner et al., 2007; Staub, 2021), our findings align with the stable, reliable individual differences in eye-movement behaviour that have been well documented for free-viewing tasks (Carter & Luke, 2018; Cronin, Hall et al., 2020; Cronin, Peacock et al., 2020; Henderson & Luke, 2014; Loh et al., 2022; Luke et al., 2018; Mills et al., 2011; Rayner et al., 2007). In this context, where there is an absence of conspicuous task demands, the consistency of eye-movement behaviour within individuals suggests a role of a more stable underlying trait (i.e., a capacity or propensity for individuals to explore scenes in distinctive ways). Although state-based factors such as task may influence eye movements, this stability in patterns within individuals across different loads suggests that substantial variance in eye-movement behaviour stems from more enduring traits.

What, then, might be some factors associated with this potential trait? Given the association of eye-movement behaviour with state-based working memory loads, one potential candidate would be trait-based working memory capacity. Although Hayes and Henderson (2017) have demonstrated that visual scan patterns are predictive of working memory capacity during a scene-encoding task, it remains unclear if the reverse relationship (i.e., that working memory capacity predicts eye-movement behaviour) is true, or how generalisable such a relationship is across different task formats. Luke et al. (2018) found that working memory span interacted with eye-movement behaviour in a way that was selective to task context: for all tasks, higher working memory capacity was linked to a more compact distribution of fixation durations, while for scene viewing, higher working memory scores predicted *longer* fixations. The authors speculate that this latter finding is linked to individuals with higher working memory capacity also being able to take in more information from peripheral vision, consistent with the idea that they have larger attentional breadths, although this claim is acknowledged to be speculative. Conversely, Loh et al. (2022) did not detect a relationship between performance on OSPAN (a commonly used, well-validated and reliable measure of working memory capacity) and eye-movement metrics, it is possible that this result was due to range restriction in OSPAN performance. This study used set sizes of 3–5, rather than the usual 3–7, with a resultant ceiling effect upon OSPAN performance clearly visible in their scatterplots. Consequently, it will be important to further investigate this relationship with a measure that tests the *limits* of individuals’ capacities before rejecting an association between working memory capacity and eye-movement behaviour.

### Attentional breadth: No effect of working memory load

In contrast to the findings for metrics of exploratory breadth, attentional breadth was not found to be affected by working memory load in this study. There are two possible explanations for this finding: firstly, that working memory load genuinely does not affect attentional breadth, and secondly, that methodological issues may have precluded observation of an effect that in fact exists. Both of these possibilities are considered in turn here.

There is a suite of studies whose findings are consistent with the conclusion that working memory load broadens attentional breadth (Ahmed & de Fockert, 2012a, b; Caparos & Linnell, 2010; Li et al., 2019; Linnell & Caparos, 2011; Zhang & Luck, 2015). However, several studies suggest that there may be moderating factors that determine when such effects are observed and when they are not (Hoar & Linnell, 2013; Li et al., 2019). For example, Hoar and Linnell (2013) found that when participants were shown hierarchical figures under varying degrees of working memory load, performance varied as a function of exposure duration. Under conditions where exposure was terminated only by participant response, a bias toward global perception of the figures was observed under low but *not* high working memory load. Conversely, when presentation durations were limited, there was no effect of working memory load, with the traditionally observed bias towards global perception remaining unchanged across load conditions. In addition, some studies have failed to observe an effect of working memory in most or all of the conditions tested: in Yao et al. (2020): over a series of nine experiments, manipulation of working memory load only appeared to modulate attentional breadth in one sub-experiment. Thus, it is not a foregone conclusion that working memory load consistently modulates attentional breadth.

Another issue that potentially undermines the strength of support for the notion that working memory load broadens attentional breadth is that much of this evidence relies on the flanker paradigm as the measure of attentional breadth (Ahmed & de Fockert, 2012a; Caparos & Linnell, 2010; Li et al., 2019; Linnell & Caparos, 2011; Zhang & Luck, 2015). The flanker paradigm appears to gauge multiple processes, including response inhibition and conflict resolution (Hübner & Töbel, 2019; Takezawa & Miyatani, 2005; Xie et al., 2017; Zhang & Luck, 2015), in addition to being considered a measure of attentional breadth (Sanders & Lamers, 2002; White et al., 2011). Response inhibition is known to degrade under higher working memory load (Boucher et al., 2021; Nyberg et al., 2009); one theoretical model (Roberts & Pennington, 1996) suggests that inhibitory control and working memory draw upon a common pool of executive functioning resources, such that as load increases, inhibitory ability decreases. This means that when an effect of working memory load on the magnitude of flanker effects is observed, it is unclear to what extent it reflects changes in other factors such as response inhibition versus attentional breadth. Finally, the attentional breadth aspect of the flanker paradigm relies on the *ability* to set a small breadth of attention that excludes the distractors surrounding the target, rather than *preference* for a particular breadth of attention; if much of the previous literature has focussed on ability measures, it might be that ability but not preference is impacted by working memory load.

Altogether, against this backdrop of somewhat equivocal support for the notion that working memory load broadens attentional breadth, it is possible that what we have observed here is a genuine instance of attentional breadth’s imperviousness to working memory load. However, it is also possible that there was an effect of working memory load on attentional breadth but we were unable to observe it for a methodological reason. One potential explanation is that when participants were completing the Navon task under the working memory loads, rather than truly multitasking (i.e., completing both tasks simultaneously), participants may have *alternated* between the tasks: calculating and enunciating the next count number, and then returning their attention to the Navon task. An alternation strategy would have added multiple sources of noise to RTs for the Navon task: firstly, participants may have multitasked on some trials but not others, while secondly, on trials where they alternated between tasks, participants are likely to have varied in how long the calculation of the next count number took them (compare the ease of calculating the backwards count from 603 to 600, versus 687 to 684). These added sources of variance in Navon task RTs are likely to have obscured the true amount of time required to respond to and identify the Navon target letter on each trial, meaning that information about how attentional breadth was affected by working memory load was not reflected in these RTs. (Note that the free-viewing task was not vulnerable to this kind of issue: as no concrete task demand was imposed at the time of scene viewing, results would not have been affected by conscious alternation between two different tasks in the same way.) The null finding obtained in this study must therefore be treated with caution: future research might require participants to update the contents of working memory in ways that do not encourage alternation between tasks that should be completed simultaneously, or alternatively investigate the relationship between attentional breadth and a more stable trait such as working memory capacity, which can be assessed separately from attentional breadth.

### Comparing effects of load on spatial deployments of attention

One possible interpretation of the results from this study is that spatial aspects of both attentional breadth and exploratory breadth are largely unaffected by working memory load, while temporal aspects of attentional deployment are affected. The covariance of attentional breadth and eye-movement behaviour has been acknowledged as an important topic (Luke et al., 2018; Rayner, 2009), but it is relatively understudied. The available evidence suggests that individual differences may also exist in the covariance of attentional breadth and eye-movement behaviour (Gaspar et al., 2016; Nuthmann, 2013; Wu & Wolfe, 2022), and in light of this study’s findings, subsequent research may benefit from distinguishing exploratory *breadth* and exploratory *dynamics* when investigating this covariance. Here, load affected the temporal dynamics of eye movements, and it remains possible that temporal aspects of attentional breadth (such as the time taken to resize attention) are similarly affected. Indeed, Goodhew (2020b) has found that individuals with high working memory capacity are *less* efficient at the infrequent contraction of attentional breadth when most trials require a broad breadth of attention. This suggests a more nuanced relationship between working memory functioning and attentional deployment than a positive correlation between capacity and superior task performance: read alongside findings from Luke et al. (2018) that indicate longer fixation durations for high-capacity individuals, one possibility is that these individuals use a *persistence* strategy for deployments of attention, whereby they tend toward exhaustive engagement with a single focus and suppression of irrelevant distractors, rather than a *flexibility *orientation, which predisposes the individual towards modification of the focus of spatial attention (Goodhew, 2020b; Hommel, 2015). Future research could attempt to shed light on some of the nuances in this relationship by comparing the temporal dynamics of attentional breadth resizing with temporal metrics of eye movements, as well as how these processes interact with state- and trait-based factors such as working memory load and capacity respectively.

## Conclusion

In conclusion, understanding and measuring spatial deployments of attention are vital steps towards modelling real-world functioning. However, the relationship between these spatial deployments and working memory load requires further investigation, particularly regarding the manipulation of information held in working memory. This study found that increasing load associated with updating and manipulation of information held in working memory affected the temporal dynamics of exploratory behaviour: individuals held fixations longer on average under higher load, fixation durations became more variable with increasing load, and scan-path lengths shortened under higher load. Conversely, the spatial aspects of eye-movement exploration did not change: exploratory breadth was not altered by increasing load. Similarly, attentional breadth was not altered by increasing load, although the possibility of methodological explanations for this finding means that strong conclusions about the relative impact of load on these two kinds of spatial deployment cannot be drawn at this stage. Intriguingly, metrics derived from eye-movement data all appeared to indicate substantial individual differences in exploratory behaviour over and above any impacts of loading working memory. This finding sets the stage for further research investigating predictors of these individual differences in exploratory behaviour, particularly working memory capacity, as well as an examination of whether *temporal* aspects of attentional breadth such as resizing are similarly affected by load. One important account to test further will be *persistence* versus *flexibility* orientation in attentional deployments: higher working memory capacity may be associated with a persistence orientation, such that single sources of information are processed more exhaustively before initiation of subsequent deployments of attention.

### Supplementary Information

Below is the link to the electronic supplementary material.

Supplementary file1 (DOCX 34 KB)

## Data Availability

The authors have full control of all primary data, which are available in an enduring repository at: https://osf.io/uszd6/.

## Footnotes

1 Rank-biserial correlation, used as a measure of effect size for non-parametric paired-samples comparisons (Kerby, 2014).

2 All variables of interest for these analyses were normally distributed as assessed by Shapiro-Wilk tests; see OSM.

3 All variables of interest for these analyses were normally distributed as assessed by Shapiro-Wilk tests; see OSM.
