# Supplementary material for: An updating-based working memory load alters the dynamics of eye movements but not their spatial extent during free viewing of natural scenes
Source: Atten Percept Psychophys. 2023 Jul 19;86(2):503–24. doi: 10.3758/s13414-023-02741-1 (PMC10805812; doi:10.3758/s13414-023-02741-1)
Supplement: Supplementary file 1 — Supplementary file1 (DOCX 34 KB) [file 13414_2023_2741_MOESM1_ESM.docx]

**Supplementary Materials**

**Counting Compliance Analysis**

***Time Calculation***

The amount of time available for counting in each trial set was calculated separately for the scene recall task and the Navon task. The image viewing component of the scene recall task ran for a fixed length of time:

*6 images × (500ms fixation + 6000ms viewing + 1000ms ISI) =* 45 seconds

Conversely, trial set duration varied for the Navon task due to differences in participant RT. As a generalised measure, the amount of time taken for each set was approximated for each participant as:

*30 trials × (participant average RT + 500ms fixation + 1000ms ISI)*

***Data Exclusions***

For both the scene viewing task and Navon task, data were excluded if the final count number was higher than the assigned starting number, or if the provided number indicated that counting was too slow (less than 1 number every 5 seconds), or too fast (more than 3 numbers per second). For the scene viewing task, one participant’s data was excluded from count compliance analysis because their final count numbers for one working memory load block did not save correctly.

***Results***

For the scene viewing task under low working memory load, 8.1% of participants’ backward count responses were excluded following the above criteria. On average after exclusions, participants counted backwards at a rate of one number per 1.6 seconds. For the scene viewing task under high working memory load, 10.0% of participants’ backward count responses were excluded. On average after exclusions, participants counted backwards at a rate of one number per 2.3 seconds.

For the scene viewing task under low working memory load, 4.9% of participants’ backward count responses were excluded following the above criteria. On average after exclusions, participants counted backwards at a rate of one number per 2.0 seconds. For the scene viewing task under high working memory load, 14.0% of participants’ backward count responses were excluded. On average after exclusions, participants counted backwards at a rate of one number per 3.2 seconds.

Overall, across the 2 x 2 (task by working memory load) design, participants completed the backwards counting task in compliance with task instructions approximately 91% of the time.

**Normality Tests: Eye Movement Metrics**

All eye movement variables used for comparisons in the Results section were subjected to Shapiro-Wilk tests to confirm that they were normally distributed; results are reported in Table 1. The only variables for which there was a violation of the assumption of normality were memory probe accuracies under both working memory loads. Inspection of histograms reveals that this is due to a ceiling effect whereby scores were clustered at close to 100% accuracy; the comparison in the main text was therefore made using a non-parametric test.

**Table 1**

*Shapiro-Wilk Tests for Eye Movement Metrics*

| Metric | Shapiro-Wilk Test | *p* |
| --- | --- | --- |
| Mean Saccadic Amplitude |  |  |
| Low WML | 0.953 | 0.082 |
| High WML | 0.982 | 0.707 |
| SD Saccadic Amplitude |  |  |
| Low WML | 0.982 | 0.723 |
| High WML | 0.961 | 0.146 |
| Mean Fixation Duration |  |  |
| Low WML | 0.966 | 0.234 |
| High WML | 0.952 | 0.070 |
| SD Fixation Duration |  |  |
| Low WML | 0.983 | 0.754 |
| High WML | 0.983 | 0.768 |
| Exploratory Breadth |  |  |
| Low WML | 0.985 | 0.848 |
| High WML | 0.971 | 0.339 |
| Scan Path Length |  |  |
| Low WML | 0.966 | 0.226 |
| High WML | 0.97 | 0.304 |
| Memory Probe Accuracy |  |  |
| Low WML | 0.857 | **< .001** |
| High WML | 0.906 | **0.002** |

*Note. P*-values below 0.05 indicate a significant violation of normality.

**Analysis of Full Dataset without Exclusions**

These analyses were performed on the full sample with complete data for each individual participant (*n =* 43). Outcomes of all analyses remained identical with or without exclusions applied (see Results).

***Navon Task Performance***

Descriptive statistics for Navon task performance are reported in Table 2. Mean accuracy for all conditions of the Navon task under both working memory loads exceeded 98%, so analysis focused primarily on RT measures. A 2 (working memory load: low versus high) x 2 (Navon target level: local versus global) repeated measures ANOVA was performed on RTs from correct trials only. There was a significant main effect of working memory load, such that participants were faster to respond in the low-load versus the high-load condition; *F*(1, 42) = 54.20, *p* < .001, η^2^_p_ = .563. This means that the working memory load demonstrably impacted participants’ performance. There was also a main effect of target level, such that participants were significantly faster when responding to target-global trials versus target-local ones; *F*(1, 42) = 4.71, *p* = .036, η^2^_p_ = .101. Finally, there was no interaction between working memory load and target level; *F*(1, 42) = 0.36, *p* = .552, η^2^_p_ = .008. The absence of an interaction suggests that the effect of working memory load did not differ for the global and local targets.

**Table 2**

*Descriptive Statistics for Navon Task Accuracy Performance*

| Variable | Accuracy (%) | | RT (ms) | |
| --- | --- | --- | --- | --- |
|  | *M* | *SD* | *M* | *SD* |
| Low Working Memory Load |  |  |  |  |
| Global Trials | 98.6 | 1.5 | 1114 | 434 |
| Local Trials | 98.5 | 2.1 | 1146 | 439 |
| High Working Memory Load |  |  |  |  |
| Global Trials | 99.0 | 1.4 | 1842 | 785 |
| Local Trials | 99.0 | 1.9 | 1901 | 833 |

***Eye Tracking Task Performance***

To test the effect of the working memory load manipulation on participants’ memory for the images presented in the eye tracking task, memory probe scores for the lower load versus the higher load were compared with a Wilcoxon signed-rank test (a non-parametric test was used as memory probe scores were not normally distributed). This indicated that memory accuracy was poorer under the higher working memory load (*M* = 85.1%, *SD* = 8.3%) compared with under the lower working memory load (*M* = 91.7%, *SD* = 7.8%); *z* = -4.06, *p* < .001, *r_rb_* = .795. This suggests that our working memory load manipulation was effective even for performance on a relatively minimal delayed recall task, where participants had a full six seconds to passively view and memorise images for identification just minutes later.

The next series of comparisons investigated the impact of working memory load upon the four types of measures for trial-level behaviour on the eye-tracking task. The first three of these measures were the means and standard deviations of (1) *fixation duration* and (2) *saccadic amplitude*, as well as (3) *exploratory breadth* (the mean Euclidean distance of each fixation from image centre), each calculated from all individual datapoints for each participant that remained after event and trial-level screening (see Methods section for more information). The final measure, (4) *scan path length*, was calculated from the total distance in pixels covered by saccades for each trial completed by each participant. Standard deviations as well as means were considered for fixation duration and saccade amplitude to indicate if the *variability* of eye movement behaviour changed between working memory load conditions. Analyses for each metric are performed using both frequentist and Bayesian t-tests using the default priors in JASP; Bayes factors are interpreted per the guidelines cited in the main text. Results are reported in Table 3.

**Table 3**

*Descriptive Statistics and Comparisons of Trial-Level Eye Tracking Measures across Working Memory Load Conditions*

| Measure | WML | | *t* | *p* | *d* | BF_10_ |
| --- | --- | --- | --- | --- | --- | --- |
|  | Low | High |  |  |  |  |
| Fixation duration mean (ms) | 377.3 (84.2) | 416.8 (97.2) | -4.985 | **< .001** | -0.760 | 1784.47 |
| Fixation duration SD (ms) | 227.0 (63.9) | 252.1 (72.6) | -3.861 | **<.001** | -0.589 | 70.56 |
| Saccadic amplitude mean (°) | 3.56 (0.82) | 3.45 (1.02) | 1.062 | .294 | 0.162 | 0.28 |
| Saccadic amplitude SD (°) | 2.75 (0.65) | 3.02 (1.00) | -2.500 | .016 | -0.381 | 2.61 |
| Exploratory breadth (pixels) | 182.9 (48.1) | 179.2 (47.4) | 0.637 | .527 | 0.097 | 0.20 |
| Scan path length (°) | 46.84 (16.47) | 40.26 (18.04) | 3.704 | **<.001** | 0.565 | 46.23 |

*Note.* Descriptive statistics for each variable are reported in the form ‘*mean (SD)*’. *n* = 34 and *df* = 42 for all comparisons. Significant p-values at the *p* < .01 level are bolded. Means are calculated at sample level for fixation duration and saccadic amplitude, then SDs are calculated at participant level and averaged across sample level, hence the differences between bracketed value after means and main value for SDs; see Methods section for more information.

***Correlations between Measures: Potential Individual Differences***

Based on the linear mixed-model analysis reported in the main body, correlations were calculated between the working memory loads for each of the key eye movement measurements derived from the free viewing recall task. While the experiment was powered for group-level rather than individual-level analyses, an exploratory analysis was performed where the correlations between eye tracking indices at each level of working memory load are reported in Table 4. These correlations show high degrees of correspondence between individuals’ eye movement behaviour in the two working memory load conditions, even though they were engaged in different tasks.

**Table 4**

*Correlations between Eye Tracking Behavioural Measures under Low and High Working Memory Loads*

| Measure | *ρ* |
| --- | --- |
|  |  |
| Fixation duration mean (ms) | .85 |
| Fixation duration SD (ms) | .81 |
| Saccadic amplitude mean (°) | .75 |
| Saccadic amplitude SD (°) | .70 |
| Exploratory breadth (pixels) | .68 |
| Scan path length (°) | .78 |

*Note.* Non-parametric correlations (Spearman’s *ρ*) are reported due to non-normality of some variables. All correlations are significant at the *p* < .001 level.
